# The effect of pregnancy on the uterine NK cell KIR repertoire

**DOI:** 10.1002/eji.201141445

**Published:** 2011-07-08

**Authors:** Victoria Male, Andrew Sharkey, Leanne Masters, Philippa R Kennedy, Lydia E Farrell, Ashley Moffett

**Affiliations:** 1 Department of Pathology and Centre for Trophoblast Research, University of CambridgeCambridge, UK; 2 Department of Cell and Molecular Biology, Imperial College LondonLondon, UK

**Keywords:** KIR, Mucosa, NK, Stroma, Uterus

## Abstract

The major leukocyte population in the decidua during the first trimester of pregnancy consists of NK cells that express receptors capable of recognizing MHC class I molecules expressed by placental trophoblast. These include members of the killer immunoglobulin-like receptor (KIR) family, the two-domain KIR (KIR2D), which recognize HLA-C. Interactions between decidual NK (dNK) cell KIR2D and placental HLA-C contribute to the success of pregnancy and dNK cells express KIR2D at higher frequency than peripheral NK (pNK) cells. Thus, they are biased toward recognizing HLA-C. In order to investigate when this unusual KIR repertoire appears, we compared the phenotype of NK cells isolated from non-pregnant (endometrium) and pregnant (decidua) human uterine mucosa. Endometrial NK (eNK) cells did not express KIR2D at a higher level than matched pNK cells, so the bias toward HLA-C recognition occurs as a response to pregnancy. Furthermore, HLA-C expression was upregulated on uterine stromal cells as the mucosa transformed from endometrium to decidua at the onset of pregnancy. As uterine NK (uNK) cells can mature from NK precursors and acquire KIR expression in utero, the pregnancy-specific bias of uNK cells toward HLA-C recognition could arise as developing uNK cells interact with uterine stromal cells, which express higher levels of HLA-C during pregnancy.

## Introduction

NK cells are the major leukocyte population in the decidua during the first few months of pregnancy and are also present in the non-pregnant endometrium [1–3]. NK cells are sparse in the proliferative phase of the menstrual cycle, but following ovulation, in the secretory phase, they proliferate, enlarge and become granulated. If fertilization occurs, the NK cells continue to proliferate in the decidua during the first trimester of pregnancy. If fertilization does not occur, they die 24–48 h before menstruation and are shed with the menses. Uterine NK (uNK) cells have cytolytic granules, but unlike their peripheral counterparts are poorly cytotoxic [4–6]. Instead, their function is likely to be to sense and respond to trophoblast, leading to regulated trophoblast invasion and spiral artery remodeling [7, 8]. In order to do this, mechanisms must exist for uNK-cell recognition of trophoblast and uNK, like peripheral blood NK (pNK) cells, express a variety of receptors, including those specific for MHC class I molecules. These are of particular interest as invasive extravillous trophoblast (EVT) has a unique complement of ligands for NK receptors: the non-classical class I molecules HLA-E and -G and the classical HLA-C [9]. CD94/NKG2A binds to HLA-E and is expressed by the majority of decidual NK (dNK) cells [10, 11]. LILRB1 is expressed by approximately 20% of dNK cells and is capable of binding to all MHC class I molecules, but has particularly high affinity for HLA-G [12].

HLA-C is the only polymorphic MHC molecule expressed by trophoblast and is the major ligand for members of the killer immunoglobulin-like receptor (KIR) family. KIRs also show considerable polymorphism in both number of KIR genes and allelic variation at individual KIR loci. KIRs specific for HLA-C can encode inhibitory (KIR2DL) or activating (KIR2DS) receptors that can discriminate between two groups of HLA-C allotypes (C1 and C2) based on a dimorphism at position 80. In general, KIR2DL1/S1 bind to C2 allotypes and KIR2DL2/3 bind to C1 allotypes, although KIR2DL2 also binds to C2 allotypes with low afffinity [13–17].

Genetic and in vitro data suggest that the combination of maternal KIR and fetal HLA-C genotypes affects the depth of trophoblast invasion [7, 18–20]. dNK cells express KIR2D specific for HLA-C at a higher frequency and level than pNK cells, meaning that they are especially equipped to recognize HLA-C [21, 22]. This bias is lost over the course of gestation so that by the second trimester the dNK-cell KIR repertoire is similar to that of pNK. Recently, we have shown that immature (stage 3) NK cells, capable of differentiating to mature NK cells in vitro, are present in the uterine mucosa, both before and during pregnancy [23]. Since immature NK cells do not express any receptors for MHC class I, it is likely that uNK acquire their KIR repertoire within the uterine microenvironment. Thus, factors present in the endometrium, decidua, or both, may affect uNK-cell receptor acquisition.

If factors common to the endometrium and decidua (that is, to the uterine microenvironment in general) mediate the acquisition of the uNK-cell KIR-repertoire, we would expect both endometrial NK (eNK) and dNK cells to display a similar bias toward HLA-C recognition. If, on the other hand, the bias arises in response to trophoblast- or pregnancy-specific factors, it would only be present in dNK, and not eNK cells. Here, we report that the KIR repertoire of eNK cells and dNK cells differs, with the bias toward HLA-C recognition only arising during pregnancy. Moreover, HLA-C expression by uterine stromal cells increases as they decidualize at the onset of pregnancy, suggesting that the acquisition of the KIR bias toward HLA-C recognition may be mediated by stromal cells in utero.

## Results

### Phenotype of eNK cells

**Figure 1:**
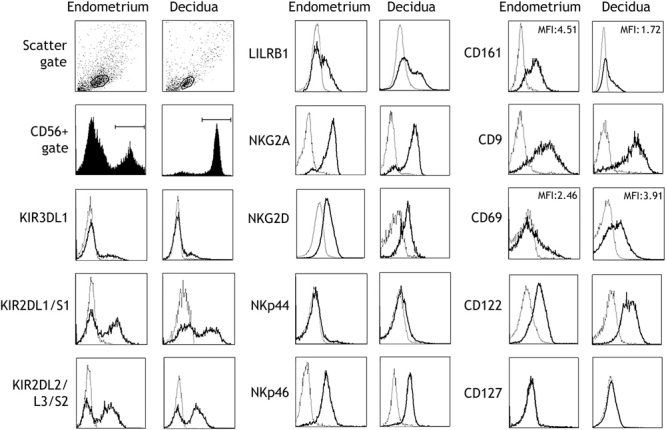
Phenotype of eNK and dNK cells. Freshly isolated preparations of endometrial and decidual leukocytes were gated on scatter and CD56^+^ cells. The gated cells were examined for expression of the markers indicated. The test stains are indicated by the bold lines; isotype control staining by dotted lines. For decidua, each panel represents at least three independent samples. For endometrium, the number of samples stained and their clinical details are given in Table 2. For CD161 and CD69, the mean MFI of all samples examined are indicated in the upper right corner. For other markers, MFI did not differ.

The phenotype of dNK cells is already well established (Table 1), but there have been few similar studies using FACS on CD56^+^ eNK. Therefore, we first compared the phenotype of freshly isolated CD56^+^ eNK with that of dNK (Fig. 1). All NK cells, both in the endometrium and in the decidua, expressed the activating receptors NKG2D and NKp46. By contrast, NKp44 was expressed by neither eNK nor dNK. CD161 (NKRP1) was expressed by all eNK but at a reduced level by dNK, while CD69, which is a marker of activation, was expressed at a lower level by eNK than dNK. For both of these antigens, there was no overlap between the endometrial and decidual sets of MFIs, but using non-parametric statistical testing it is not possible to say that the differences observed were statistically significant. All uNK cells expressed the IL-2 receptor beta chain CD122, whereas none expressed the IL-7 receptor alpha chain CD127. Thus, with the exceptions of CD161 and CD69, which may differ in their level of expression between eNK and dNK, overall the uNK-cell phenotype was very similar in the non-pregnant and pregnant uterus.

**Table 1:** Phenotype of eNK as defined by previous studiesa)

	pNK	pNK	eNK	dNK	References
CD56	Dim	Bright	Superbright	Superbright	[2, 38]
CD16	+	–	–	–	[2, 36, 38]
2B4	+	+	ND	+	[26, 45, 56]
CD9	−	−	+	+	[38, 44]
CD94	−/+	(−)/+	(−)/+	(−)/+	[11, 35, 37]
CD69	−	−	ND	+	[44]
CD117	−	Dim	ND	Dim	[60]
KIR	−/+	−	−/+	−/+	[21, 22, 35, 37]
NKG2D	+	+	+	+	[36, 43, 59]
NKp30	−	−	−	Disputed	[7, 26, 36, 43, 45]
NKp44	−	−	−	Disputed	[7, 26, 36, 43, 45]
NKp46	+	+	+	+	[7, 26, 36, 43, 45]
Perforin	+	Dim	+	+	[5, 44, 61]
Granzyme	+	Dim	+	+	[5, 44, 61]

a) − no expression of marker, + all cells positive, −/+ some cells negative, some positive, (−)/+ some cells negative but most positive, ND; not done.

**Figure 2:**
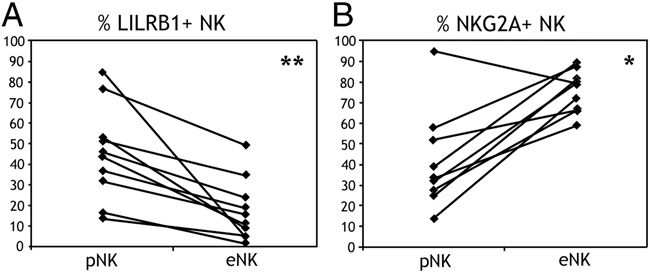
eNK cells have a lower frequency of LILRB1 but a higher frequency of NKG2A expression than their peripheral counterparts. The frequency of receptor expression in pNK is shown on the left of each graph; frequency in eNK cells is shown on the right. Matched pairs are joined by a line. Frequency of (A) LILRB1 and (B) NKG2A expression are shown. ^*^p<0.05, ^**^p<0.01, Wilcoxon signed rank test. All data points obtained are represented on the plots.

The inhibitory NK receptors that will recognize the trophoblast MHC molecules HLA-G and HLA-E are LILRB1 and CD94/NKG2A respectively, and these receptors were expressed at similar frequencies by eNK and dNK (Fig. 1). There is still no consensus about whether KIR2DL4 recognizes HLA-G and which NK cells express this KIR [24]. Using a variety of monoclonal antibodies, we have never been able to detect KIR2DL4 expression either at the cell surface or in endosomes of uNK cells (our unpublished observations). We compared the frequency of LILRB1 and NKG2A expression on eNK with that of pNK taken from the same woman at the same time. NKG2A was expressed at a significantly higher frequency by eNK compared with pNK (*n*=9; Fig. 2B) whereas LILRB1 was expressed at significantly lower frequency by eNK compared to pNK (*n*=10; Fig. 2A).

### The uNK cell bias toward recognizing HLA-C is not present before pregnancy

**Figure 3:**
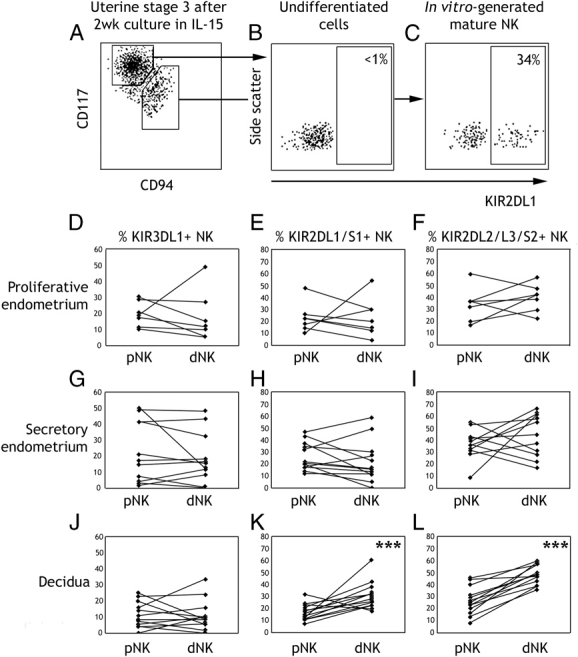
KIR2D are more frequently expressed in dNK cells than pNK cells, but there is no difference between pNK cells and eNK cells. (A) Immature (stage 3) NK cells were isolated from the decidua and cultured for 2 wk in 1 nM IL-15. Expression of CD117 and CD94 at the end of culture is shown. (B) KIR expression by those immature NK cells that had not differentiated by the end of the culture period. (C) KIR expression by the in vitro-generated mature NK cells. (A–C) are representative of three independent experiments. (D–L) KIR expression in freshly isolated CD56^+^ uNK cells compared with that of freshly isolated matched CD56^+^ pNK cells: frequency of KIR expression in pNK cells is shown on the left of each graph, frequency in uNK cells is shown on the right, matched pairs are joined by a line. Expression of (D, G, J) KIR3DL1, (E, H, K) KIR2DL1/S1 and (F, I, L) KIR2DL2/L3/S2 are shown in proliferative endometrium (D–F), secretory endometrium (G–I) and decidua (J–L) respectively. ^***^p<0.001, Wilcoxon signed rank test.

We have recently reported the presence of immature NK cells, which do not express any receptors for MHC class I, in the uterine mucosa. These cells are able to progress to a mature NK phenotype in vitro, acquiring CD94 and losing CD117 expression [23]. To see whether immature NK cells in the uterus can also acquire KIR expression, we cultured them for 2 wk with recombinant human IL-15 and examined their expression of CD117, CD94 and KIR2DL1/S1 by FACS. Those cells that acquired CD94 also expressed some KIR (5–34%) whereas those that did not acquire CD94 remained KIR^−^ (Fig. 3A–C). Therefore, the hormonally driven microenvironment in the uterine mucosa, where these immature NK cells acquire KIRs, is likely to influence the uNK cell KIR repertoire.

To determine whether the KIR repertoire was altered by pregnancy, we next compared the expression of KIRs by uNK from either endometrium or decidua (Fig. 1) with that of matched pNK. Taking either endometrial (*n*=18) or decidual (*n*=13) isolates and peripheral blood leukocytes, CD56^+^ cells were examined for expression of KIR3DL1, KIR2DL1/S1 and KIR2DL2/L3/S2. The analysis of data as matched pairs, which has not been done in previous studies, takes into account the marked differences in KIR expression between individuals [25]. Similar to our earlier findings, the frequency of NK cells positive for either KIR2DL1/S1 (specific for HLA-C2) or KIR2DL2/L3/S2 (KIR2DL2 and -2DL3 recognize HLA-C1) was significantly higher in the decidua than matched blood (Fig. 3K and L). In contrast, there was no significant difference in frequency of KIR3DL1^+^ NK cells (specific for HLA-Bw4; HLA-B is not expressed by trophoblast cells) (Fig. 3J). Similar results were observed in both KIR AA donors, and individuals who expressed activating KIRs. This provides additional confirmation that dNK are more equipped to recognize HLA-C molecules than their peripheral counterparts [21, 22].

The high frequency of KIR2D expression on uNK could either be due to a pregnancy-specific factor or occur as a result of factors common to the mucosal microenvironment in utero both before and during pregnancy. Therefore, we examined the KIR repertoire of eNK cells from either the proliferative (*n*=7) or the secretory (*n*=11) phase of the menstrual cycle, compared with matched pNK. As expected, eNK expressed KIR3DL1 with a frequency that was not significantly different from that of pNK (Fig. 3D and G). Unlike dNK, though, there was no significant difference between expression of KIR2D in blood and eNK cells, at any phase of the menstrual cycle (Fig. 3E–F, H–I). We conclude that the bias toward KIR recognition of HLA-C is only a feature of uNK cells in pregnancy. Consistent with this, the frequency of KIR2D expression by dNK was higher than that by eNK, although this was only significant for KIR2DL2/L3/S2.

### The KIR repertoire of pNK and uNK cells are different before pregnancy

**Figure 4:**
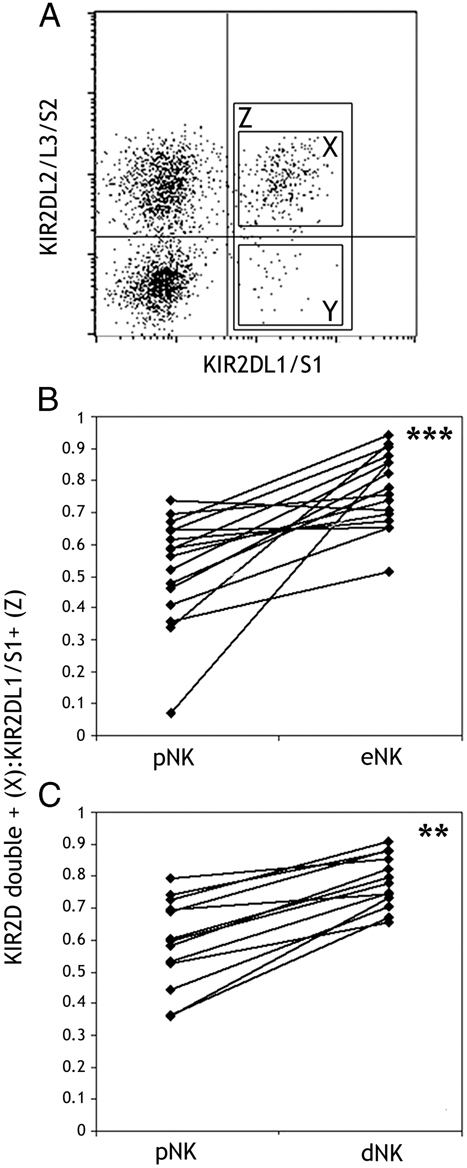
NK cells expressing KIR2DL1/S1 but not KIR2DL2/L3/S3 are rarer in the uterus than matched blood. (A) Preparations of leukocytes freshly isolated from endometrium, decidua or peripheral blood were gated on CD56^+^ and examined for KIR2DL1/S1 and KIR2DL2/L3/S2 expression. Gate Z contains all KIR2DL1/S1 positive cells; gate X KIR2D double-positive cells (KIR2DL1/S1^+^KIR2DL2/L3/S3^+^); gate Y contains KIR2DL1/S1 single-positive cells. The proportion of KIR2DL1/S1^+^ NK cells that are KIR2D double positive is quantitated by dividing the number of cells in gate X by gate Z. Comparisons between (B) blood and endometrium and (C) blood and decidua are shown. ^**^p<0.01, ^***^p<0.001, Wilcoxon signed rank test. (B, C) All data points obtained are represented on the plots.

An additional feature observed was that, compared with pNK, there were very few uNK cells expressing KIR2DL1/S1 alone (Fig. 4A gate Y). This means that most KIR2DL1/S1^+^ NK cells also expressed KIR2DL2/L3/S2 (Fig. 4A gate X). The number of these KIR2D double-positive cells (KIR2DL1/S1^+^KIR2DL2/L3/S2^+^; Fig. 4A gate X) as a proportion of the total number of KIR2DL1/S1^+^ cells (Fig. 4A gate Z) was consistently higher in uNK than in matched pNK, both in endometrium and decidua, suggesting that it is due to factors independent of pregnancy (Fig. 4B and C).

### Placental HLA-C does not affect the dNK-cell KIR repertoire

Following implantation, HLA-C^+^ EVT invade into the decidua, where they intermingle with uNK cells. It is possible that the skew toward KIR2D expression could arise as a response to the higher levels of HLA-C expression on EVT, compared with other cells in the mucosa [20]. In this case, we might expect KIR expression on uNK to be skewed not just toward recognition of HLA-C in general, but toward recognition of the specific HLA-C (group 1 or 2) expressed by the trophoblast in each pregnancy. To examine this possibility, we extracted genomic DNA from placental villi and typed the samples for HLA-C1/C2 in an attempt to correlate fetal HLA-C genotype with the expression of cognate KIRs on maternal uNK cells. The presence of trophoblast HLA-C2 or -C1 did not increase the proportion of dNK-expressing KIR2DL1/S1 or KIR2DL2/L3/S2, respectively (Supporting Information Fig. 1A and B). As well as recognizing HLA-C1, KIR2DL2 also binds to HLA-C2 alleles with low affinity, and this could have confounded the analysis [16, 17], but even when KIR2DL2^+^ individuals were excluded from the analysis, no correlation between placental HLA-C and maternal expression of KIRs was found. Thus, the pregnancy-specific bias of uNK toward recognition of HLA-C does not appear to be a response to HLA-C expression by trophoblast.

### Soluble factors present in the pregnant uterus cannot recapitulate the dNK-cell bias toward HLA-C recognition

A soluble factor produced by trophoblast cells might account for the dNK-cell bias toward HLA-C recognition. However, there was no difference in KIR expression by eNK cultured either in the presence or absence of trophoblast-conditioned medium (Supporting Information Fig. 2A–C). Another possibility is that IL-15, which is expressed in the uterine mucosa in a progesterone-dependent manner and is known to affect KIR expression by NK cells, could account for the unique dNK-cell KIR repertoire [26–29]. To investigate this, we cultured eNK in either the presence or absence of 2.5 ng/mL IL-15 and this did increase KIR2D expression, compared with culture without cytokines. However, IL-15 also promoted the maintenance of KIR3DL1 expression and thus is unlikely to account for the dNK-cell bias toward HLA-C recognition (Supporting Information Fig. 2D–F).

### HLA-C levels are increased on decidual compared with endometrial stromal cells

**Figure 5:**
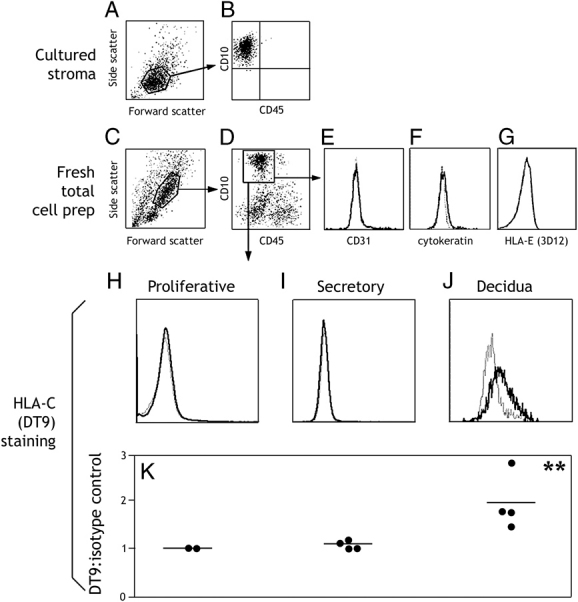
HLA-C is more highly expressed by decidual than endometrial stromal cells. (A) Cultured decidual stromal cells were gated by scatter characteristics and (B) examined for their expression of CD10 and CD45. Total fresh decidual and endometrial cell preparations were gated by (C) scatter characteristics and on (D) CD10^+^CD45^−^ cells. These cells were negative for (E) the endothelial cell marker CD31, (F) the epithelial cell marker cytokeratin 7, and (G) HLA-E was undetectable on the cell surface. Geometric mean fluorescence intensity when staining with the HLA-C-specific antibody DT9 on stroma from (H) proliferative endometrium, (I) secretory endometrium and (J) decidua is shown. Antibody staining is indicated by the bold lines; isotype control staining is indicated by dotted lines. (K) The scatter plot shows all values obtained (*n*=2 proliferative samples and *n*=4 each of secretory and decidual). ^**^p<0.01, comparing endometrial (proliferative and secretory together) and decidual data points, Mann–Whitney *U* test.

The MHC class I molecules expressed by bone marrow stromal cells are important for the acquisition of the Ly49 repertoire by developing mouse NK cells [30]. The same may to be true of KIR in humans and certainly in vitro NK differentiation does require stromal feeder cells for robust KIR acquisition [31]. Therefore, a change in HLA-C expression by uterine stromal cells could account for the pregnancy-specific skew toward HLA-C recognition in uNK. To test this hypothesis, we stained uterine stromal cells (identified by scatter and as CD10^+^CD45^−^ cells: Fig. 5A–F) with the monoclonal antibody DT9, which is specific for HLA-C on primary cells [32, 33]. DT9 does not bind to beads bearing any common alleles of HLA-A or -B, except for HLA-A80 and -B73 [32, 33], but can bind to cell lines that overexpress HLA-E [32–34]. However, we observed no staining of uterine stromal cells with the HLA-E-specific monoclonal antibody 3D12 by flow cytometry (Fig. 5G). Thus, DT9 staining is representative of HLA-C expression on uterine stromal cells. HLA-C was undetectable on stromal cells in the endometrium (Fig. 5H and I) but levels of expression were significantly higher on decidual cells (Fig. 5J and K; *p*<0.01), indicating that HLA-C levels increase as stromal cells decidualize.

## Discussion

Humans and other menstruating primates are unique among mammals in that their uterine mucosa commences its transformation from endometrium to decidua before implantation occurs, in the secretory phase of every menstrual cycle. NK cells are a feature of decidua and, during this phase of the cycle, eNK proliferate vigorously under the influence of progesterone. No side-by-side comparison of receptor expression by eNK and dNK has previously been undertaken; here, we characterize these eNK cells in detail.

Similar to dNK and in agreement with previous reports (Table 1), CD56^bright^ eNK are uniformly positive for NKG2D and NKp46, uniformly negative for NKp44, mostly positive for NKG2A and a substantial proportion express KIR [35–38]. Thus, using these phenotypic markers, eNK are identical to dNK cells. We also show that a fraction (mean 18.2%) of eNK express the inhibitory receptor LILRB1, comparable to the 20% of LILRB1^+^ dNK [12]. In addition, eNK are uniformly positive for CD161, although staining for CD161 was stronger on eNK than on dNK. The ligand for CD161, LLT1, is expressed by trophoblast [39, 40], suggesting that CD161 may be downregulated by dNK in the presence of its ligand. The unique NK cells found in the pregnant mouse uterus are also negative for CD161 (NK1.1) [41]. Consistent with a comparison of eNK and dNK-cell transcriptomes, the activation marker CD69 is more highly expressed by dNK than eNK, indicating that uNK cells have become activated during pregnancy [42]. We found that NKp44 was not expressed on dNK, in agreement with one previous report [43] but in conflict with others [7, 44, 45]. In at least one of the latter studies, dNK were cultured before analysis with IL-2, which is known to induce NKp44 expression [46]. Thus, eNK and dNK are phenotypically very similar, so that many of the unique phenotypic features of uNK are present before pregnancy.

The NK lineage may comprise two sublineages, one of which is CD127^+^ and IL-7-dependent, while the other is CD122^+^ and IL-15-dependent [47]. It is therefore interesting that uNK express CD122 (IL-2Rβ, a component of the IL-15 receptor) but not CD127 (IL-7Rα), in keeping with the known hormonal regulation of IL-15 in the uterine mucosa [26, 27]. Most evidence thus points to uNK belonging to the IL-15-dependent lineage. That uNK are able to develop in response to IL-15 is evident from our report that IL-15 is sufficient to induce maturation of the immature NK cells present in endometrium and decidua [23]. Here, we demonstrate that IL-15 also induces KIR expression on these NK precursors as they differentiate to mature NK cells in vitro. When this occurs in vivo, the uterine microenvironment will affect uNK-cell receptor repertoire acquisition. The unusual KIR repertoire in dNK, which is skewed toward recognizing HLA-C, is not present in eNK. Thus, uNK cells are only biased toward recognizing HLA-C after pregnancy has begun. However, some subtle differences in the uNK-cell KIR repertoire are present even before pregnancy: NK cells expressing only KIR2DL1/S1, and not also expressing KIR2DL2/L3/S2, are very scarce in the uterus compared with the periphery, and this is true of both eNK and dNK. KIR2DL2/3 is expressed before KIR2DL1, so one possible way to account for this observation is that KIR2D negative uNK are not permitted to acquire KIR2DL1 until they have first acquired KIR2DL2/L3 [17, 48–50]. This explanation also resonates with observations of KIR acquisition by donor NK cells in hematopoietic stem cell transplant recipients over time [49, 50]. At early time points after transplantation, very few cells are positive for only KIR2DL1/S1, so this abundance of KIR2D double-positive NK cells may be a feature of NK cells that have only recently matured and acquired KIR.

Trophoblast expresses high levels of HLA-C [20] and we therefore considered that the pregnancy-specific bias toward HLA-C recognition might be caused by the selective expansion of those NK cells that could recognize trophoblast. We were unable to correlate frequency of KIR expression with the presence of cognate HLA-C on trophoblast, but the numbers in this study are small and a trend may still emerge with larger numbers. Indeed, expression of KIR2DL1/S1 by CD3^+^ T cells in term decidua is affected by fetal HLA-C2 expression [51]. Term and first trimester decidua differ in a number of important ways: in particular, the dNK-cell skew toward HLA-C recognition is lost over the course of pregnancy [22] and is entirely absent at term [51]. In this context, whether T cells from first trimester decidua have the ability to recognize HLA-C by either TCR or KIRs is unknown. In culture, we were unable to recapitulate the skew toward KIR2D expression observed in dNK, either by treating eNK with soluble factors produced by trophoblast, or with IL-15. Therefore, none of these factors seem likely to be responsible for the dNK-cell bias toward HLA-C recognition.

The explanation we favor to account for this bias is that, during decidualization, stromal cells upregulate surface expression of HLA-C. Our results using the HLA-C-specific monoclonal antibody DT9 confirm the puzzling report that *HLA-C*, but not *-A* or *-B*, transcripts are increased in secretory compared with proliferative endometrium [52]. Uterine stromal cells express progesterone receptors and undergo a number of changes in response to progesterone [53, 54]. The observations reported here, together with those previously published on HLA transcription over the menstrual cycle [52], suggest that HLA-C expression is likely to be progesterone-responsive in these cells. The lack of HLA-A and -B-specific antibodies does make it difficult to definitively confirm that the increase in uterine stromal HLA-C expression during pregnancy does not also encompass HLA-A and -B molecules. However, when cells from HLA-A2^+^ individuals were examined with the HLA-A2-specific antibody BB7.2, we observed no differences between endometrial and decidual stroma (not shown). uNK are firmly associated with stromal cells by a variety of integrin/matrix interactions and, by electron microscopy, the two are in intimate contact in vivo [55, 56]. Furthermore, the effect of uterine stroma on developing NK cells is likely not to be limited to cell–cell interactions: conditioned medium from decidual stromal cells has been shown to induce a uNK-cell-like phenotype in pNK, and this is TGFβ-dependent [57]. Therefore, there is the opportunity for extensive cross-talk between uNK and uterine stromal cells that can affect uNK-cell development and repertoire acquisition.

In summary, we find that, although both eNK and dNK express KIRs, it is only dNK that are consistently biased toward recognition of HLA-C. Expression of HLA-C on uterine stromal cells increases during decidualization at the beginning of pregnancy. This could explain the pregnancy-specific bias of uNK toward HLA-C recognition, which only arises when immature NK cells in the uterus acquire their KIR repertoire in the presence of high levels of stromal HLA-C. Thus, as well as the profound effects decidualization has on stromal, glandular and vascular elements of the mucosa, it also affects uNK-cell activation and KIR repertoire.

## Materials and methods

### Primary tissue

Protocols using human tissue were approved by the Cambridge Local Research Ethics committee (study numbers 04/Q0108/23 and 08/H0305/40). Totally, 30 endometrial samples were taken by Pipelle biopsy from normally cycling women attending for tubal ligation. Samples from women who were bleeding at the time of surgery were assigned as menstrual. Other samples were designated proliferative or secretory on the basis of cycle day number and serum progesterone (Table 2). Totally, 27 decidual samples were taken from women attending for elective termination of pregnancy between 8 and 11 wk after their last menstrual period. Matched venous blood was obtained from all patients at the time of surgery.

**Table 2:** Clinical details of non-pregnant donors

Patient	Cycle day	Cycle length	Serum progesterone (ng/mL)	Phase assigned	Antigens examined
01	2	24	0.69	Menstrual	CD9, KIR
02	4	24	1.38	Menstrual	CD9, KIR
03	2	28	Not tested	Menstrual	CD9, LILRB1
04	1	28	Not tested	Menstrual	CD9. KIR
05	13	28	0.75	Proliferative	CD9, KIR
06	4	25	1.29	Proliferative	CD9, KIR, NKG2A, LILRB1
07	17	28	1.82	Proliferative	CD9, KIR, LILRB1
08	6	28	Not tested	Proliferative	CD9, KIR
09	8	28	1.06	Proliferative	CD9, KIR, LILRB1
10	9	28	0.79	Proliferative	CD9, KIR
11	8	28	0.85	Proliferative	CD9, KIR
12	7	28	0.62	Proliferative	CD9, CD69, CD161
13	27	28	7.52	Secretory	CD9, KIR
14	24	28	6.73	Secretory	CD9, KIR, NKG2A, LILRB1
15	26	28	2.45	Secretory	CD9, KIR, NKG2A, LILRB1
16	19	28	6.82	Secretory	CD9, KIR, NKG2A, LILRB1
17	20	28	11.73	Secretory	CD9, KIR, NKG2A, LILRB1
18	25	32	18.81	Secretory	CD9, KIR
19	24	32	11.35	Secretory	CD9, KIR, LILRB1
20	17	28	13.84	Secretory	CD9, KIR
21	19	28	3.9	Secretory	CD9, KIR
22	19	28	7	Secretory	CD9, KIR, NKG2A, LILRB1
23	18	28	23.69	Secretory	CD9, NKG2A
24	18	28	14.08	Secretory	CD9, NKG2A
25	14	28	28.1	Secretory	CD9, NKG2A, LILRB1
26	19	25	4.43	Secretory	CD9, KIR
27	21	28	Not tested	Secretory	CD9, CD127
28	21	28	1.63	Secretory	CD9, CD122
29	27	28	2.95	Secretory	CD9, NKG2D
30	29	31	6.98	Secretory	CD9, CD69, CD161, NKp44, NKp46

Lymphocytes were extracted from peripheral blood by layering onto Lymphoprep (Axis-Shield) and taking the interface. Cells were extracted from uterine samples as described [58]. Briefly, pieces of uterine tissue were washed in RPMI 1640 medium (Gibco), minced and digested with collagenase V (Sigma) (for decidua: 10 mg/mL, 1 h, 37°C; for endometrium: 0.6 mg/mL, 12 h, room temperature). The suspension was passed through a series of cell strainers to 40 μm, and layered onto Lymphoprep to enrich for leukocytes. The interface was taken, washed in PBS and stained immediately, or cultured for up to seven days in RPMI 1640 medium, plus antibiotics, 10% FCS and any other indicated additions. All uterine samples were double stained for CD56 and CD9 (Fig. 1) and those with any CD56^+^CD9^−^ cells present discarded as this indicates contamination by maternal blood leukocytes [44]. Decidual stromal cells were separated from the leukocytes and cultured as described [58]. Immature (stage 3) dNK cells were extracted and cultured as described [23].

### Flow cytometry

Cells were suspended in 0.2 mg/mL human γ globulins (Sigma) (15 min, 4°C) to block non-specific binding before incubation with fluorochrome-conjugated antibody (30 min, 4°C). Directly conjugated antibodies were: CD56, KIR2DL1/S1 clone EB6, LILRB1 clone HPE-F1 (Beckman Coulter); CD9, CD94 (clone 131412), KIR2DL2/L3/S2 (BD); CD10, CD31, CD45, CD56, CD69, CD117 (clone 104D2), CD122, KIR3DL1 clone DX9, NKG2D, NKp44 (Biolegend); cytokeratin 7 (Dako), which was detected intracellularly using FIX & PERM (ADG); CD127 (eBioscience); NKp46 (R&D). NKG2A (clone Z199, Beckman Coulter), CD161 (BD), HLA-C (DT9, a kind gift of Veronique Braud) and HLA-E (clone 3D12, eBioscience) expression was examined by indirect staining. Cells were blocked in human γ globulins and incubated with primary antibody (30 min, 4°C), then washed and incubated with PE anti-mouse (Sigma) (30 min, 4°C). Following a further wash, cells were blocked in 0.1 mg/mL mouse γ globulins (Sigma) (15 min, 4°C) and stained with directly conjugated antibody as above. Data were collected on a BD FACScalibur, with at least 10 000 events collected through the gate of interest.

### Genotyping of KIR and HLA-C

KIR and HLA-C were typed from genomic DNA as described [18, 19]. Briefly, the individual genes were genotyped by PCR for their presence or absence with sequence-specific primers using two pairs of primers per gene or allele. KIR genes typed were 3DL1, 3DS1, 2DL1, 2DL2, 2DL3, 2DL5, 2DS1, 2DS2, 2DS3, 2DS4, 2DS5 and 2DP1. Typing for HLA-C was performed using a similar approach, which allowed all known HLA-C group C1 alleles to be distinguished from C2 alleles.

### Statistical analysis

The frequencies of KIR expression in matched blood and uterine NK cells were compared using the Wilcoxon signed rank test. The Mann–Whitney *U* test was used to make comparisons between non-matched data sets. The results with *p*<0.05 were considered significant.

## Abbreviations

dNK cell: decidual NK cell
eNK cell: endometrial NK cell
EVT cell: extravillous trophoblast cell
KIR cell: killer immunoglobulin-like receptor cell
KIR2D cell: the two-domain KIR cell
pNK cell: peripheral NK cell
uNK cell: uterine NK cell
