# Supplementary material for: The effect of pregnancy on the uterine NK cell KIR repertoire
Source: Eur J Immunol. 2011 Jul 8;41(10):3017–27. doi: 10.1002/eji.201141445 (PMC3262970; doi:10.1002/eji.201141445)
Supplement: Supplementary file 1 [file eji0041-3017-SD1.pdf]

European Journal of Immunology

Supporting Information

for

DOI 10.1002/eji.201141445

The effect of pregnancy on the uterine NK cell KIR repertoire

Victoria Male, Andrew Sharkey, Leanne Masters,
Philippa R. Kennedy, Lydia E. Farrell and Ashley Moffett

Supporting Information

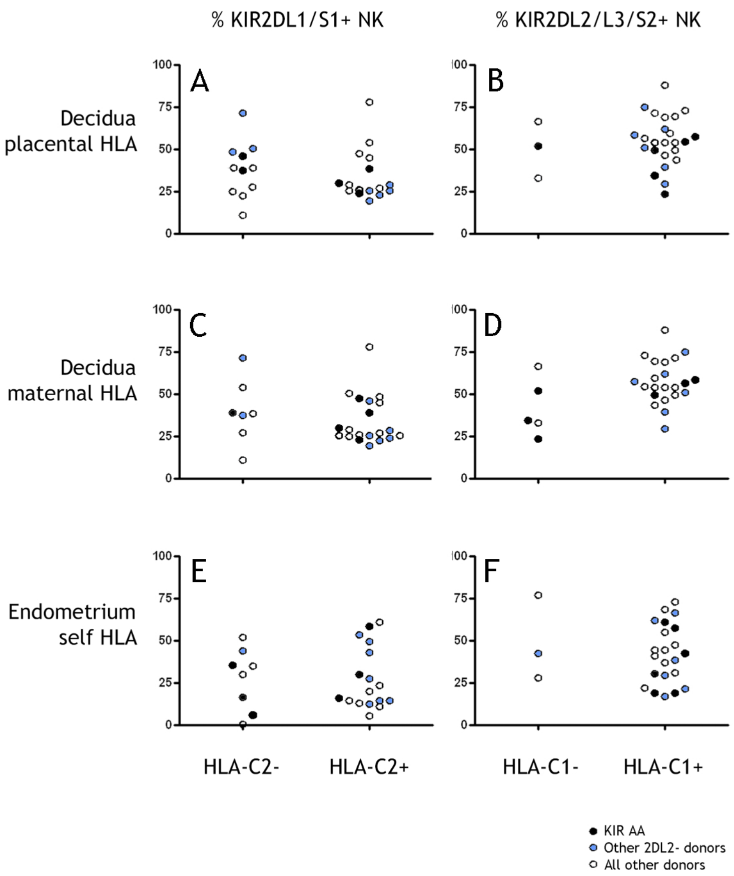

Supplementary Figure 1: *Neither self nor fetal HLA-C status affects KIR expression*
Frequency of two-domain KIR expression on uNK cells was correlated with presence or absence of its cognate HLA-C ligand on trophoblast (A and B), or expressed by the female donor (C and D in pregnancy, E and F non-pregnancy). No comparisons were significant, even when KIR2DL2+ individuals were excluded.

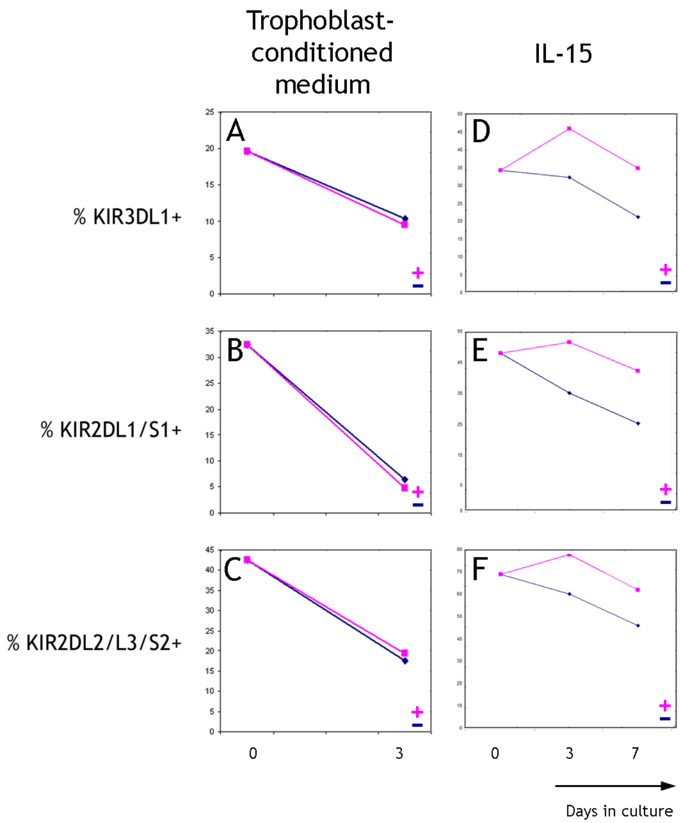

Supplementary Figure 2: *Neither culture with trophoblast-conditioned medium nor with IL-15 recapitulates uNK bias towards HLA-C recognition*
A – C: KIR expression was examined on fresh eNK cells, and after three days culture either in the presence (pink line) or absence (blue line) of 50% trophoblast-conditioned medium. D – E: KIR expression on fresh eNK cells, and after three or seven days culture either in the presence (pink line) or absence (blue line) of 2.5ng/ml human re-combinant IL-15.
